# Impact of postoperative daily image-guided intensity-modulated radiotherapy on overall and local progression-free survival in patients with oral cavity cancer

**DOI:** 10.1186/s12885-016-2165-9

**Published:** 2016-02-23

**Authors:** Chen-Hsi Hsieh, Pei-Wei Shueng, Li-Ying Wang, Yu-Chuen Huang, Li-Jen Liao, Wu-Chia Lo, Yu-Chin Lin, Le-Jung Wu, Hui-Ju Tien

**Affiliations:** Division of Radiation Oncology, Department of Radiology, Far Eastern Memorial Hospital, No.21, Sec. 2, Nanya S. Rd., Banciao Dist., New Taipei City, 220 Taiwan; Department of Medicine, School of Medicine, National Yang-Ming University, Taipei, Taiwan; Institute of Traditional Medicine, School of Medicine, National Yang-Ming University, Taipei, Taiwan; Department of Radiation Oncology, National Defense Medical Center, Taipei, Taiwan; Oriental Institute of Technology, New Taipei City, Taiwan; School and Graduate Institute of Physical Therapy, College of Medicine, National Taiwan University, Taipei, Taiwan; Department of Medical Research, China Medical University Hospital, Taichung, Taiwan; School of Chinese Medicine, China Medical University, Taichung, Taiwan; Department of Otolaryngology, Far Eastern Memorial Hospital, Taipei, Taiwan; Division of Medical Oncology and Hematology, Department of Internal Medicine, Far Eastern Memorial Hospital, Taipei, Taiwan; Physical Therapy Center, National Taiwan University Hospital, Taipei, Taiwan

**Keywords:** Helical tomotherapy, IGRT, Image-guided, Intensity-modulated radiation therapy, Oral cavity cancer

## Abstract

**Background:**

We compared the outcome of patients who received non-image-guided intensity-modulated radiotherapy (IMRT) with those who received helical tomotherapy (HT), a daily image-guided radiotherapy (IGRT), after surgery for oral cavity cancer (OCC).

**Methods:**

During the period November 2006 to December 2013, a total of 152 postoperative OCC patients underwent either IMRT (*n* = 79) or daily IGRT (*n* = 73) 4 to 6 weeks after surgery. Patients in the IMRT group received 6 MV photon beams to 7 fields and those in the IGRT group received daily fractions of 1.8 or 2 Gy on five consecutive days.

**Results:**

Patients who received daily IGRT had higher 5-year overall survival than those who received IMRT (87 % versus 48 %, *p* = 0.015). The local progression-free survival rate was also higher in patients who received IGRT (85 % versus 58 %, *p* = 0.006). More patients in the IGRT group completed the package of overall treatment time in ≤ 13 weeks and completed their course of radiation therapy in ≤ 8 weeks than patients in the IMRT group (89 % versus 68 %, *p* = 0.002; 84 % versus 58 %, *p* = 0.001), respectively. The rate of local failure in the primary tumor area was 24.0 % in the IMRT group and 6.8 % in the IGRT group. Among patients with primary local failure, the marginal failure rate was 52.6 % in the IMRT group and 0 % in the IGRT group.

**Conclusions:**

For patients with locally advanced OCC, postoperative IGRT results in better overall survival, better local progression-free survival, less marginal failure and shorter overall treatment time than postoperative non-image-guided IMRT.

## Background

Intensity-modulated radiation therapy (IMRT) is safe and efficacious in the adjuvant setting for oral cavity cancer (OCC) [1–4]. Compared with conventional radiotherapy, IMRT provides better normal tissue protection by producing highly conformal doses of radiation to targets and sharp dose gradients between targets and surrounding critical structures [5]. Nonetheless, patients with head and neck cancer tend to experience changes in soft tissue and body weight throughout the course of radiation therapy, which have an impact on the delivered dose [6]. Zeidan et al. [7] found that more than 10 % of patients with head and neck cancer who underwent imaging every other treatment day had setup errors of ≥ 5 mm. Image-guided IMRT provides an excellent mechanism to account and correct for patient setup errors. Den et al. [8] demonstrated that the use of image-guided radiotherapy (IGRT) for position verification and alignment resulted in improvement in margin shrinkage due to decreased radiation exposure to normal tissues.

Helical tomotherapy (HT) is an IGRT system comprising megavoltage computed tomography (MVCT) that provides precise delivery of photons to a specific target region, thereby sparing critical organs from exposure to radiation and reducing the toxic effects associated with radiation therapy [3, 9–11]. HT has also been demonstrated to have better target volume dose conformity and homogeneity than IMRT [12–14]. However, there is a lack of consensus in the literature as to whether IGRT results in better survival than non-IGRT.

In this study we compared the clinical outcome of patients who received non-image-guided IMRT with those who received daily IGRT after surgery for oral cavity cancer.

## Methods

### Patient characteristics

During the period December 2006 to December 2013, 189 patients with OCC underwent IMRT without image guidance or daily HT at the Far Eastern Memorial Hospital. Of those patients, 152 individuals without a history of disease recurrence after surgery who received radiotherapy with or without concurrent chemotherapy were retrospectively enrolled. All patients were initially evaluated by a multimodality treatment team consisting of an otolaryngologist, oral surgeon, medical oncologist, and radiation oncologist. Staging investigations included a complete medical history and physical examination, fiber-optic endoscopic evaluation, complete blood cell counts, liver function tests, chest X-ray, preoperative magnetic resonance imaging (MRI) of the head and neck region, and a dental evaluation. Bone scans and computed tomography (CT) of the chest and abdomen were obtained whenever possible before the beginning of treatment. All tumor specimens were staged according to the tumor-node-metastasis (TNM) staging system (AJCC cancer staging manual, 6^th^ edition).

### Radiation therapy

Radiation therapy with or without concurrent chemoradiation therapy (CCRT) was initiated 4 to 6 weeks after surgery using 6 MV photon beams and a 7-field IMRT plan or daily HT with a simultaneous-integrated boost or sequential techniques comprising 1.8 or 2 Gy fractions on five consecutive days. The choice of dose was made at the discretion of the primary oncologist and the choice of treatment modality was left up to the patients. In Taiwan, only non-IGRT is covered by the National Health Insurance system. Therefore, patients who chose to undergo HT had to pay out-of-pocket for the procedure. Target regions and normal structures were contoured using the Pinnacle 3 Treatment Planning System (Philips Healthcare, Madison, Wisconsin, USA). The preoperative MR images were retrieved on a Pinnacle workstation and fused with the CT images by rigid image registration in all patients to contour the postoperative flap and confirm the location of the preoperative gross tumor to overcome the possibility of miss-contouring of the gross tumor due to structural changes caused by surgery.

### Delineation of target volumes

The clinical target volumes (CTVs) were determined according to the incidence and location of metastatic neck nodes from various head and neck subsites as previously reported [3, 15]. Briefly, CTV1 was defined as the area encompassing the preoperative gross tumor and postoperative flap plus a 0.8- to 1-cm margin, which included the resection bed with soft-tissue invasion by the tumor or extracapsular extension (ECE) by metastatic neck nodes truncating air, and uninvolved bones. CTV2 was defined as a high-risk subclinical area primarily including the pathologically uninvolved cervical lymph nodes, deemed as elective nodal regions, or prophylactically treated neck areas. CTV3 was designated as a low-risk area of potential subclinical disease. To account for organ motion and patient setup errors, the CTVs were used to construct the planning target volumes (PTVs). PTV1 and PTV2 included CTV1 and CTV2 plus a margin of 3 mm for HT and 5 mm for IMRT, while PTV3 included CTV3 plus a margin of 5 mm for HT and 7 mm for IMRT. PTV1 consisted of 60–66 Gy in 30–33 fractions; high-risk OCC patients received 64–66 Gy and intermediate-risk OCC patients received 60 Gy. PTV2 comprised 59.4–60 Gy in 30–33 fractions and PTV3 consisted of 46–54 Gy in 23–33 fractions.

Additionally, no more than 20 % of the PTV received more than 110 % of its prescribed doses, and no more than 1 % of any PTV received less than 93 % of its prescribed doses. The dose constraints for organs at risk (OARs) were as follows: (1) a maximum dose of 54 Gy for brainstem; (2) a maximum dose of 45 Gy for spinal cord; (3) a maximum dose of 45 Gy for optic chiasm and optic nerve; (4) a mean dose of < 30 Gy for bilateral parotid glands with a median dose of < 26 Gy. For parotid glands with a volume larger than 20 mL, the median dose was < 20 Gy; (5) a mean dose of 2/3 of glottic larynx < 50 Gy (6) a mean dose of < 50 Gy for inner ear; and (7) a maximum dose of 70 Gy for mandible.

Patients treated by HT underwent daily IGRT via MVCT. The daily MVCT images were fused with the original treatment planning prior to administration of each fraction based on soft tissue and bony structures. After automatic registration, the position was corrected manually in order to align the PTV.

### Chemotherapy

Studies have shown that close or positive resection margin, extracapsular spread, perineural invasion, lymphovascular space involvement, primary tumor stage T3-4 and two or more positive lymph nodes are significant predictors of poor overall survival and local control in patients with head and neck cancer [16–18]. Patients with any of those prognostic factors underwent concurrent chemotherapy. Concurrent chemotherapy comprising weekly intravenous administration of cisplatin (30 mg/m^2^) plus fluorouracil (5-FU, 425 mg/m^2^) and leucovorin (30 mg/m^2^) was received by 85 % (*n* = 67) of patients in the IMRT group and 89 % (*n* = 65) of patients in the HT group (Table 1).

**Table 1:** Patient characteristics

	IMRT (non-IGRT) (No. = 79)	HT (IGRT) (No. = 73)	*P* value
Variable		No. of patients (%)	
Age (years)			
Median	48	52	0.398
Range	29–78	24–78	
Gender			
Male	78 (98.7 %)	70 (95.9 %)	0.274
Female	1 (1.3 %)	3 (4.1 %)	
Subsite			
Oral tongue	25 (31.6 %)	35 (47.9 %)	0.348
Buccal mucosa	34 (43.0 %)	25 (34.2 %)	
Alveolar ridge	8 (10.1 %)	7 (9.6 %)	
Retromolar trigone	5 (6.3 %)	3 (4.1 %)	
Floor of the mouth	2 (2.5 %)	2 (2.7 %)	
Hard palate	2 (2.5 %)	1 (1.4 %)	
Lip	3 (3.8 %)	0	
Resection-margin status			
Close or positive	39 (49.4 %)	42 (57.5 %)	0.313
Negative	40 (50.6 %)	31 (42.5 %)	
Extracapsular spread			
Positive	13 (16.5 %)	19 (26.0 %)	0.148
Negative	66 (83.5 %)	54 (74.0 %)	
Perineural involvement			
Positive	49 (62.0 %)	60 (82.2 %)	0.006
Negative	30 (38.0 %)	13 (17.8 %)	
Lymphovascular space involvement			
Positive	31 (39.2 %)	46 (63.0 %)	0.003
Negative	48 (60.8 %)	27 (37.0 %)	
Pathology stage:			
Tumor stage			
Stage I	6 (7.6 %)	8 (11.0 %)	0.532
Stage II	12 (15.2 %)	12 (16.4 %)	
Stage III	18 (22.8 %)	15 (20.5 %)	
Stage IVA	43 (54.4 %)	38 (52.1 %)	
Primary tumor stage			
T1	14 (17.7 %)	13 (17.8 %)	0.768
T2	22 (27.8 %)	25 (34.2 %)	
T3	18 (22.8 %)	11 (15.1 %)	
T4a	25 (31.6 %)	24 (32.9 %)	
Regional lymph node stage			
N0	39 (49.4 %)	38 (52.1 %)	0.302
N1	15 (19.0 %)	7 (9.6 %)	
N2a	8 (10.1 %)	6 (8.2 %)	
N2b	14 (17.7 %)	20 (27.4 %)	
N2c	3 (3.8 %)	1 (1.4 %)	
N3	0	1 (1.4 %)	
Adjuvant concurrent chemotherapy			
Yes	67 (84.8 %)	65 (89.0 %)	0.442
No	12 (15.2 %)	8 (11.0 %)	
RT dose			
Median (range)	66 Gy (59.4–72 Gy)	66 Gy (60–70.2 Gy)	0.304
POTT			
≤13 weeks	46 (58.2 %)	61 (83.6 %)	0.001
>13 weeks	33 (41.8 %)	12 (16.4 %)	
OTTRT			
≤8 weeks	54 (68.4 %)	65 (89.0 %)	0.002
>8 weeks	25 (31.6 %)	8 (11.0 %)	

*Abbreviations: ECOG Performance Status* Eastern Cooperative Oncology Group Performance Status, *HT* helical tomotherapy, *IGRT* image-guided radiotherapy, *IMRT* intensity-modulated radiotherapy, *non-IGRT* non-image-guided radiotherapy, *OTTRT* overall treatment time of radiotherapy, *POTT* package of overall treatment time

### Definition of relapse and delineation of locoregional failure

When available, image studies delineating the site of locoregional failure were fused with the treatment planning CT scan. Otherwise, anatomic landmarks were used to determine the failure site. Failure was defined as infield if >95 % of the volume of the recurrent tumor fell within the CTV, marginal if 20–95 % of the volume was within the CTV, and out of field if 20 % fell within the CTV [19].

### Follow-up

All patients were evaluated at least once a week during radiotherapy. Upon completion of radiation, patients were evaluated every 3 months for the first 2 years. At each follow-up visit, a fiber-optic endoscopic examination and palpation of the neck were performed as part of the physical examination. Post-treatment MRI of the oral cavity and neck was done 1, 3, and 6 months after completion of radiotherapy. Acute toxicities (occurring < 90 days after initiation of radiotherapy) and late toxicities (occurring > 90 days after initiation of radiotherapy) were defined and graded according to the Common Terminology Criteria for Adverse Events v3.0 (CTCAE v3.0). The earliest date of detecting grade 3 or worse toxicity was recorded.

### Statistical methods

Descriptive statistics (mean, median, proportions) were calculated to characterize the patients, diseases, treatment features and toxicities after treatment. The overall survival (OS), disease-free survival (DFS), locoregional progression-free survival (LRPFS), local progression-free survival (LPFS), regional progression-free survival (RPFS) and metastasis-free survival (MFS) rates were estimated using the Kaplan-Meier product-limit method and log-rank tests [20]. Significant variables in the univariate analyses were included in a multivariate regression model to identify the most important factors associated with outcome. Cox proportional-hazards analysis was used to determine the relative contribution of various factors to outcome. Freedom from local and regional progression was defined as the absence of a primary tumor and regional lymph nodes on physical examination and on any radiographic examination (CT and MRI). Durations were calculated from the date of pathologic proof. All analyses were performed using the statistical package SPSS for Windows (Version 20.0, IBM Corporation, Armonk, NY, USA).

All procedures performed in studies involving human participants were in accordance with the ethical standards of the institutional and/or national research committee and with the 1964 Helsinki declaration and its later amendments or comparable ethical standards. The need for informed consent was waived by the Institutional Review Board of the Far Eastern Memorial Hospital (FEMH-IRB- 104008-E) and retrospective data were collected after receiving approval from the Institutional Review Board of the Far Eastern Memorial Hospital (FEMH-IRB- 104008-E).

## Results

### Patient characteristics

One hundred and forty-eight men and four women were included in the study. The median age was 52 years (range, 24–78 years). As seen in Table 1, the dominant subsites of oral cancer in the IMRT and HT groups were oral tongue (32 % and 48 %) and buccal mucosa (43 % and 34 %). There were no significant differences in oral cancer location between the two groups of patients. There were also no significant differences between the IMRT and HT groups in resection margin status, extracapsular spread, pathological stage, receipt of concurrent chemotherapy, or RT dose. However, the percentage of patients with lymphovascular space involvement (LVSI) was significantly higher among HT-treated patients than among those who received non-image-guided IMRT (63 % versus 39 %, *p* = 0.003). Also, the percentage of patients with perineural involvement (PNI) was significantly higher in the HT group than in the IMRT group (82 % versus 62 %, *p* = 0.006). (Table 1) The median dose of radiation in both groups was 66 Gy. In this study, the total time from surgery to completion of therapy (package of overall treatment time, POTT) was dichotomized into >13 weeks and ≤ 13 weeks and the overall duration of radiation therapy (overall treatment time of radiation therapy (OTTRT)) was dichotomized into > 8 weeks and ≤ 8 weeks. We found that significantly more patients in the HT group completed the package of overall treatment time in ≤ 13 weeks and completed their course of radiation therapy in ≤ 8 weeks than patients in the IMRT group (POTT, 84 % vs 58 % (*p* = 0.001); OTTRT, 89 % vs 68 %, (*p* = 0.002)).

### Treatment outcomes

**Fig. 1:**
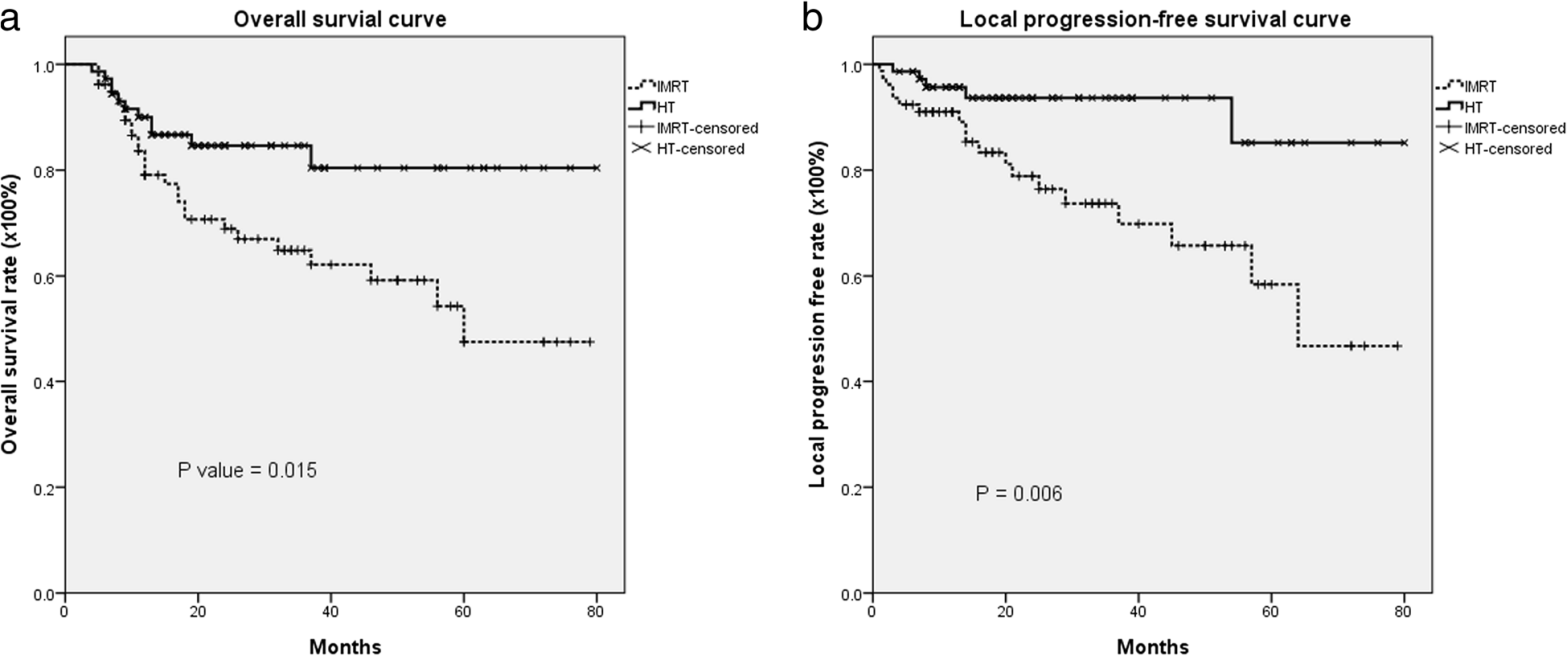
The actuarial 5-year Kaplan–Meier survival estimates. **a** Overall survival curve; **b** Local progression-free survival curve for postoperative oral cavity cancer patients treated with intensity-modulated radiation therapy (IMRT) or helical tomotherapy (HT), with or without concurrent chemotherapy

**Fig. 2:**
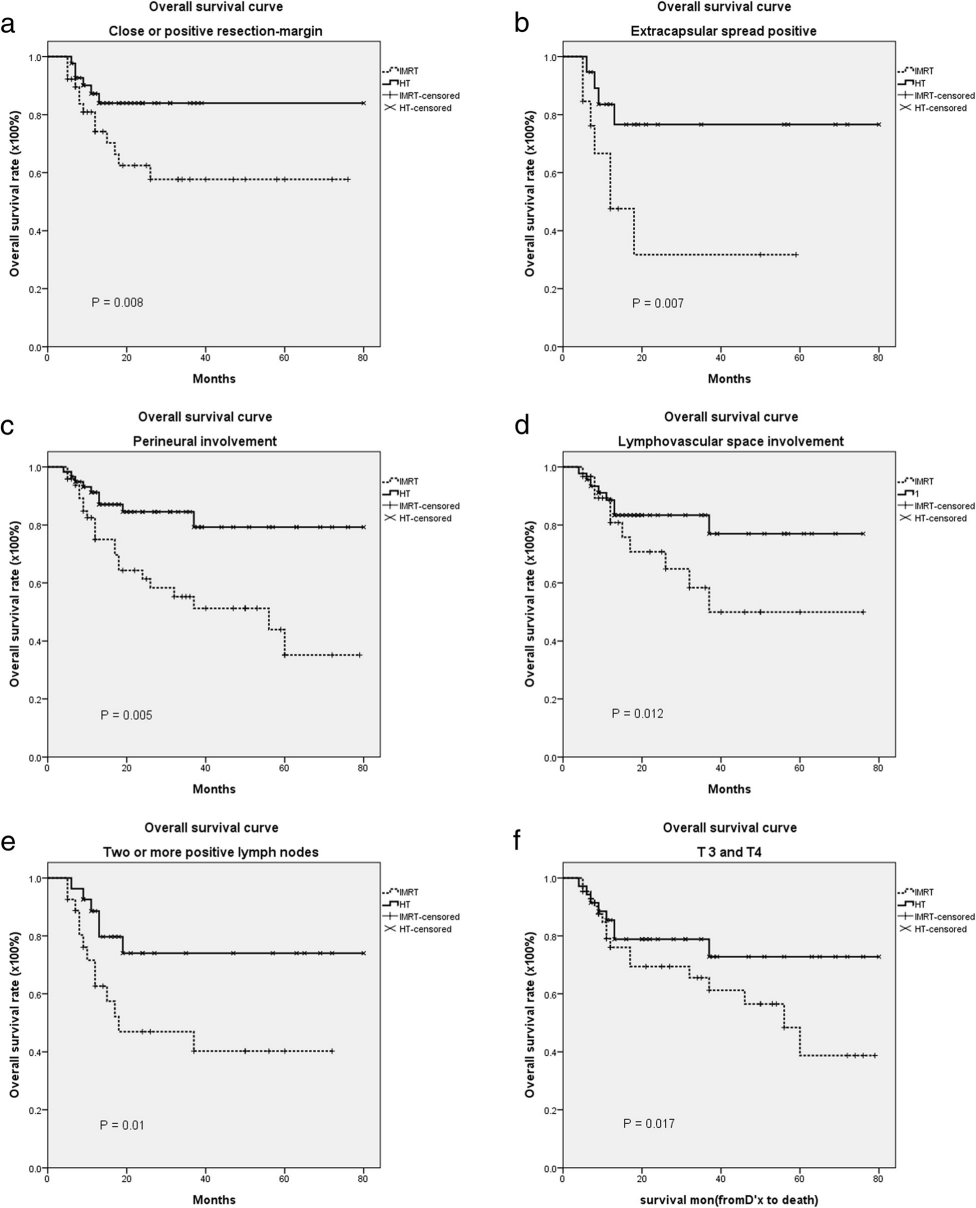
The actuarial 5-year Kaplan–Meier curves for overall survival curve according to risk factors in patients with postoperative oral cavity cancer patients treated with intensity-modulated radiation therapy (IMRT) or helical tomotherapy (HT), with or without concurrent chemotherapy. **a** positive resection-margin; **b** positive extracapsular spread (ECE); **c** positive perineural invasion (PNI); **d** positive lymphovascular space involvement (LVSI); **e** two or more positive lymph nodes; **f** T3,4

**Fig. 3:**
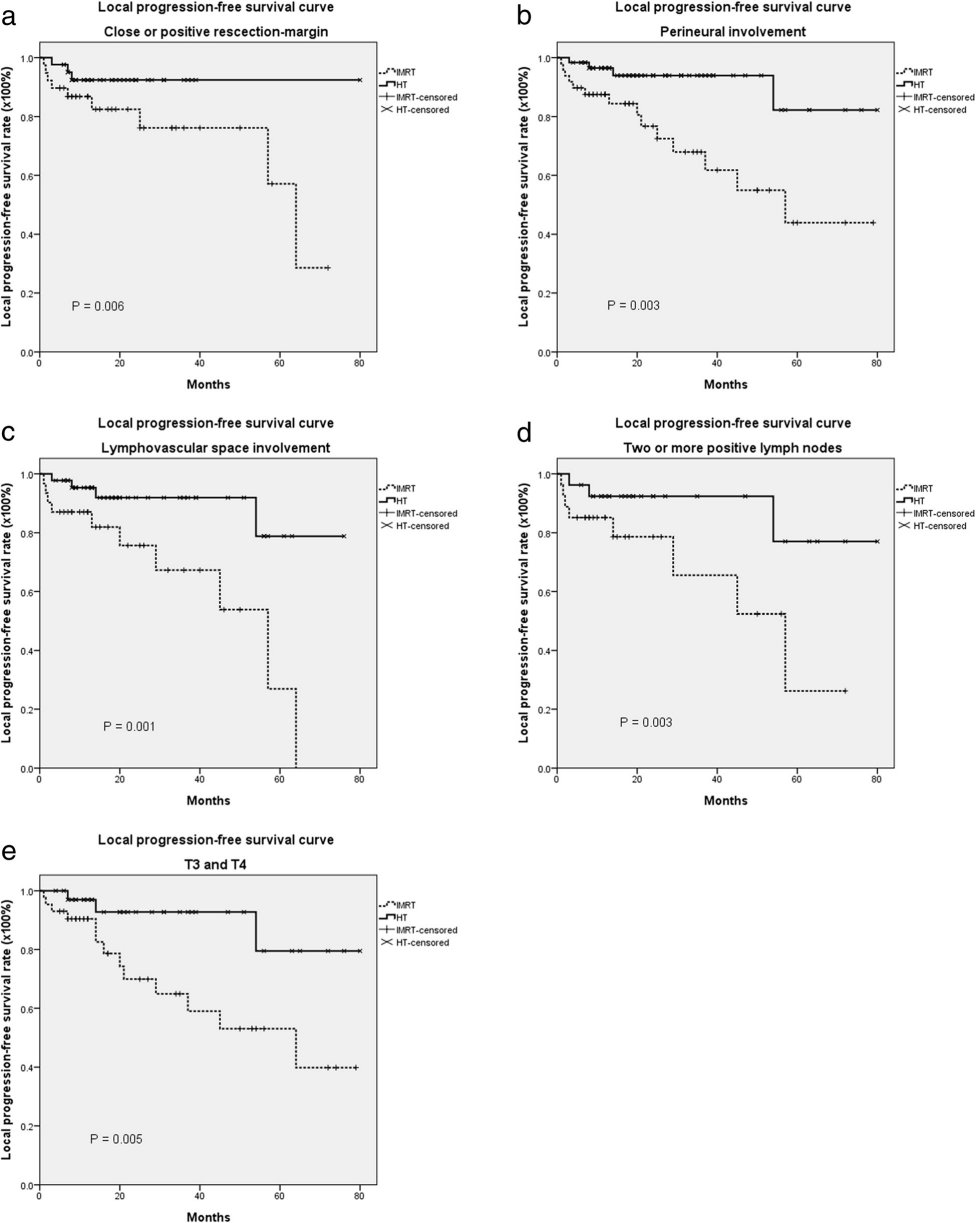
The actuarial 5-year Kaplan–Meier estimates of local progression-free survival according to risk factors in postoperative oral cavity cancer patients treated with intensity-modulated radiation therapy (IMRT) or helical tomotherapy (HT), with or without concurrent chemotherapy. **a** positive resection-margin; **b** positive perineural invasion (PNI); **c** positive lymphovascular space involvement (LVSI); **d** two or more positive lymph nodes; **e** T3,4

The median follow-up was 60 months (range, 4 to 80 months). The actuarial 5-year overall survival (OS), disease-free survival (DFS), locoregional progression-free survival (LRPFS), local progression-free survival (LPFS), regional progression-free survival (RPFS) and metastasis-free survival (MFS) rates in each group are listed in Table 2. There were no significant differences in the majority of those outcome measures; however, the rates of overall survival and local progression-free survival were significantly higher in the HT group than in the non-image-guided IMRT group (Table 2 and Fig. 1a-b). Also, Kaplan–Meier estimates of 5-year OS and 5-year LPFS in patients with prognostic factors such as positive resection margin, positive extracapsular spread, positive perineural invasion, positive lymphovascular space involvement, two or more positive lymph nodes, or tumor stage T3/T4, revealed that patients who received HT had better overall survival (Fig. 2a to f) and local progression-free survival (Fig. 3a to e) than patients who received IMRT without image guidance. Significant variables in the univariate analyses (ECE, PNI, LVSI, two or more positive lymph nodes and T3,4) were included in a multivariate regression model to identify the most important factors associated with outcome. Cox proportional-hazards analysis was used to determine the relative contribution of various factors to outcome. The results revealed that patients who underwent IGRT had significantly better 5-year overall survival rates (adjusted hazard ratio (HR) = 0.318; 95 % CI = 0.152-0.666, *p* = 0.002) and significantly better 5-year local progression-free survival than patients who underwent IMRT without image guidance (HR = 0.211; 95 % CI = 0.076–0.591, *p* = 0.003) (Table 3).

**Table 2:** The actuarial 5-year overall survival, disease-free survival, locoregional progression-free survival, local progression-free survival, regional progression-free survival and metastasis-free survival rates in the intensity-modulated radiotherapy-treated and helical tomotherapy-treated groups

Survival rate	IMRT (non-IGRT)	HT (IGRT)	95 % CI	*p* value
5-year OS	47.5 %	86.7 %	0.53 to 0.64	0.015
5-year DFS	39.3 %	73.8 %	0.40 to 0.74	0.146
5-year LRPFS	49.8 %	69.8 %	0.37 to 0.77	0.104
5-year LPFS	58.4 %	85.2 %	0.58 to 0.70	0.006
5-year RPFS	81.2 %	81.4 %	0.62 to 0.72	0.653
5-year MFS	82.6 %	80.1 %	0.62 to 0.72	0.892

*Abbreviations: DFS* disease-free survival, *HT* helical tomotherapy, *IGRT* image-guided radiotherapy, *IMRT* intensity-modulated radiotherapy, *MFS* metastasis-free survival, *non-IGRT* non-image-guided radiotherapy, *LRPFS* locoregional progression-free survival, *LPFS* local progression-free survival, *OS* overall survival, *RPFS* regional progression-free survival

**Table 3:** The 5-year overall survival rate and local-progression free survival rate of postoperative oral cavity cancer patients under prognostic factors treated with helical tomotherapy (HT, image-guided radiotherapy, IGRT) and intensity-modulated radiotherapy (IMRT, non- image-guided radiotherapy, non-IGRT)

	5-year OS				5-year LPFS			
Prognostic factors	Modality		95 % CI	*p* value	Modality		95 % CI	*p* value
	HT	IMRT			HT	IMRT		
Resection-margin	84.0 %	57.7 %	0.52 to 0.68,	0.008	92.5 %	28.6 %	0.51 to 0.77	0.006
ECE	76.6 %	31.7 %	0.39 to 0.65,	0.007	54.0 %	64.1 %	0.39 to 0.65	0.942
PNI	79.3 %	35.2 %	0.15 to 0.97	0.005	82.3 %	43.9 %	0.28 to 0.85	0.003
LVSI	77.0 %	50.0 %	0.49 to 0.64	0.012	78.8 %	0.0 %	0.54 to 0.74	0.001
Two or more positive lymph nodes	74.0 %	40.3 %	0.41 to 0.61	0.010	77.0 %	26.2 %	0.29 to 0.85	0.003
T3,4	72.8 %	38.7 %	0.39 to 0.73	0.017	79.5 %	39.8 %	0.25 to 1.02	0.005
	Hazard ratio (HR)^a^		95 % CI	*P* value	Hazard ratio (HR)^a^		95 % CI	*P* value
Modality HT vs IMRT	0.32		0.15 to 0.67	0.002	0.21		0.08 to 0.59	0.003

*Abbreviations: CI*, confidence interval; *ECE* extracapsular spread, *HT* helical tomotherapy, *IMRT* intensity-modulated radiotherapy, *LPFS* local progression-free survival rate, *LVSI* lymphovascular space involvement, *OS* overall survival rate, *PNI* perineural invasion

^a^Hazard ratios were derived from Cox proportional hazards regression after adjusting ECE, PNI, LVSI, two or more positive lymph nodes and T3,4 variables

### The local failure in the primary tumor area for both groups

The rate of local failure in the primary tumor area was 24.0 % (19/79) in the non-image-guided IMRT group and 6.8 % (5/73) in the HT group. Of the 19 patients in the IMRT group with local failure, 14 had perineural invasion only, 10 had lymphovascular space involvement only and 8 patients had concomitant perineural invasion and lymphovascular space involvement. In addition, of the 19 patients with local failure, 31.6 % (*n* = 6), had infield failure, 52.6 % (*n* = 10) had marginal failure and 15.5 % (*n* = 3) had out-of-field failure. Of the 5 patients with local failure after receiving HT, four had concomitant perineural invasion and lymphovascular space involvement. Failure was defined as infield in four of the five patients and out-of-field in one of the five patients.

### The influences of POTT and OTTRT on survival

In the non-IGRT group, the overall treatment time of radiotherapy (OTTRT) was 6.5 weeks in 18 (22.8 %) patients, 7 weeks in 33 (41.8 %) patients and 8 weeks in 53 (67.0 %) patients. In the IGRT group, the OTTRT was 6.5 weeks in 23 (31.5 %) patients, 7 weeks in 46 (63.0 %) patients and 8 weeks in 65 (89.0 %) patients. The total number of patients who received radiotherapy for ≥7 weeks was 46 (58.2 %) in the IMRT group and 27 (40 %) in the IGRT group. The total number of patients who received radiotherapy for ≥8 weeks was 25 (32 %) in the non-IGRT group and 8 (11 %) in the IGRT group.

**Fig. 4:**
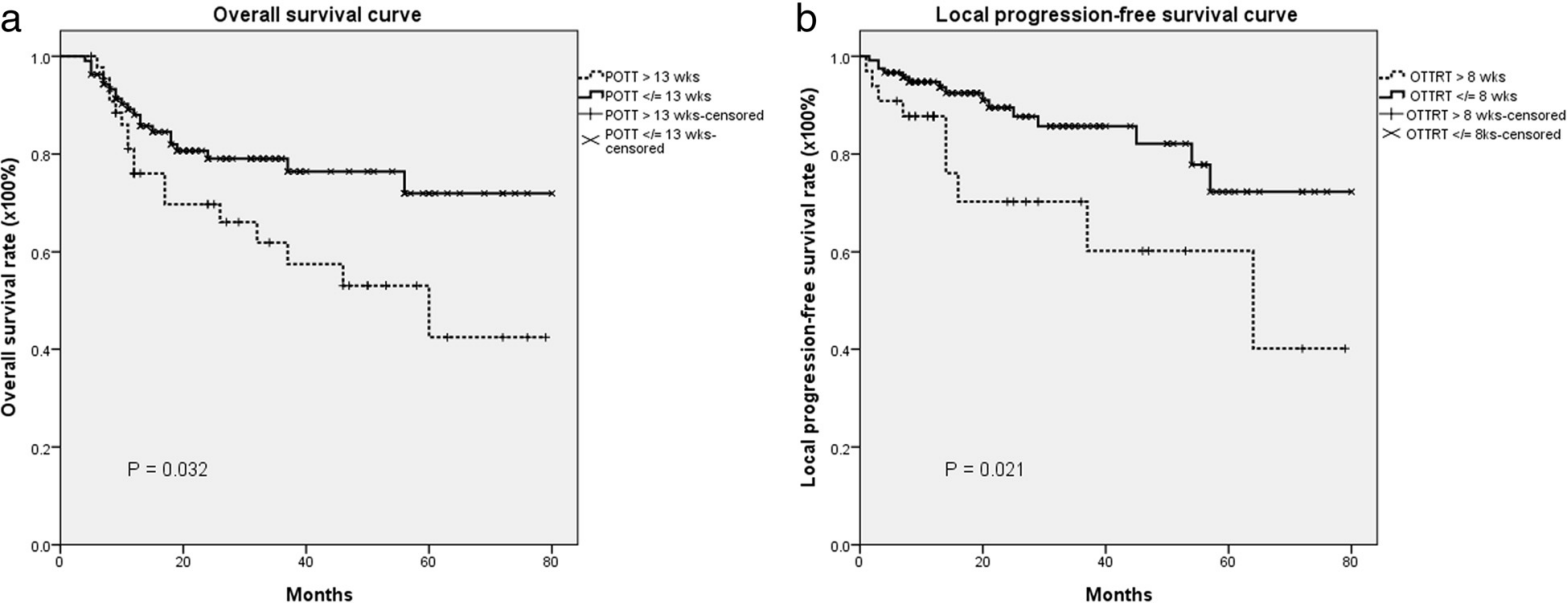
**a** The actuarial 5-year Kaplan–Meier curves for overall survival in postoperative oral cavity cancer patients treated with radiotherapy with a package of overall treatment time (POTT) less than 13 weeks or more than 13 weeks; **b** The actuarial 5-year -year Kaplan–Meier estimates of local progression-free survival in postoperative oral cavity cancer patients treated with radiotherapy with an overall treatment time of radiation therapy (OTTRT) less than 8 weeks or longer than 8 weeks

Kaplan–Meier survival estimates revealed that a POTT ≤ 13 weeks was significantly associated with better overall survival in both groups (*p* = 0.032 by the log-rank test, 95 % CI, 0.24 to 0.96) (Fig. 4a). Comparison of actuarial survival rates between both groups of patients who completed the POTT in ≤ 13 weeks revealed that patients in the IGRT group had significantly better overall survival and better local progression-free survival than patients in the non-IGRT group (85.0 % vs 58.3 % (95 % CI, 0.24 to 0.96, *p* = 0.05) and 85.8 % vs 59.1 % (95 % CI, 0.31 to 0.97, *p* = 0.015), respectively).

Kaplan–Meier survival estimates revealed that an OTTRT ≤ 8 weeks was significantly associated with better local progression-free survival than an OTTRT > 8 weeks in both groups (72.3 % *vs.* 40.1 %, 95 % CI, 0.15 to 1.12, *p* = 0.021) (Fig. 4b). Comparison of actuarial survival rates between both groups of patients with an overall duration of therapy ≤ 8 weeks revealed that patients in the IGRT group had significantly better overall survival and better local progression-free survival than patients in the non-IGRT group (85.0 % vs 58.3 % (95 % CI, 0.24 to 0.96, *p* = 0.05) and 88.0 % vs 56.2 % (95 % CI, 0.15 to 1.12, *p* = 0.025), respectively).

### Toxicities

Data on incurred toxicities in both groups of patients with or without chemotherapy are detailed in Table 4. No grade 3 xerostomia was noted in either group. The incidence of grade 2/3 body weight loss was significantly lower in the IGRT group than in the non-IGRT group (*p* = 0.004). There were no significant differences in incidence of grade 3 dermatitis (*p* = 0.180) or grade 2/3 dysphagea (*p* = 0.412) between the two groups. The constraints for critical organs in patients who underwent IGRT or non-IGRT were the same. As the plan could be accepted, the doses for the critical organs were within limitation for both techniques that might cause the similar adverse effects as non-IGRT. There were no differences in incidence of fistula formation or mucositis between the two groups. One reason for this finding might be because the mucosa is part of the CTV in patients with OCC and mucosal reactions dominate acute reactions regardless of treatment modality. The incidence rates of leucopenia (*p* = 0.007) and thrombocytopenia (*p* = 0.003) were significantly higher in patients who underwent IMRT without image guidance than in patients who underwent IGRT (Table 4).

**Table 4:** Acute toxicities in high-risk oral cavity cancer patients treated with postoperative helical tomotherapy (HT, image-guided radiotherapy, IGRT) and intensity-modulated radiotherapy (IMRT, non- image-guided radiotherapy, non-IGRT) with or without concurrent chemoradiation therapy (CCRT)

^b^Toxicity	IMRT (non-IGRT) (No. = 79)	HT (IGRT) (No. = 73)	*P* value
		No. of patients (%)	
^a^Xerostomia (Acute)			
Gr.1	54 (68.4 %)	54 (74.0 %)	0.445
Gr.2	25 (31.6 %)	19 (26.0 %)	
Gr.3	0	0	
Gr.4	0	0	
Gr.5	0	0	
Mucositis			
Gr.1	7 (8.9 %)	4 (5.5 %)	0.627
Gr.2	49 (62.0 %)	44 (60.3 %)	
Gr.3	23 (29.1 %)	25 (34.2 %)	
Gr.4	0	0	
Gr.5	0	0	
Dermatitis			
Gr.1	33 (41.8 %)	33 (45.2 %)	0.180
Gr.2	30 (47.6 %)	33 (45.2 %)	
Gr.3	16 (20.3 %)	7 (9.6 %)	
Gr.4	0	0	
Gr.5	0	0	
Body weight loss			
Gr.1	51 (64.6 %)	62 (84.9 %)	0.004
Gr.2	27 (34.2 %)	11 (15.1 %)	
Gr.3	1 (1.3 %)	0	
Gr.4	0	0	
Gr.5	0	0	
Dysphagia			
Gr.1	53 (67.1 %)	55 (75.3 %)	0.472
Gr.2	11 (13.9 %)	9 (12.3 %)	
Gr.3	15 (19.0 %)	9 (12.3 %)	
Gr.4	0	0	
Gr.5	0	0	
Fistula formation or superficial cases of skin dehiscence			
Yes	1 (1.3 %)	3 (4.1 %)	0.351
No	78 (98.7 %)	70 (95.9 %)	
Anemia			
Normal	11 (13.9 %)	15 (20.5 %)	0.507
Gr.1	58 (73.4 %)	51 (69.9 %)	
Gr.2	10(12.7 %)	7 (9.6 %)	
Gr.3	0	0	
Gr.4	0	0	
Gr.5	0	0	
Leucopenia			
Normal	14 (17.7 %)	24 (32.9 %)	0.007
Gr.1	49 (62.0 %)	25 (34.2 %)	
Gr.2	9 (11.4 %)	17 (23.3 %)	
Gr.3	5 (6.3 %)	6 (8.2 %)	
Gr.4	2 (2.5 %)	1 (1.4 %)	
Gr.5	0	0	
Thrombocytopenia			
Normal	15 (19.0 %)	31 (42.5 %)	0.003
Gr.1	59 (74.7 %)	41 (56.2 %)	
Gr.2	3 (3.8 %)	1 (1.4 %)	
Gr.3	2 (2.5 %)	0	
Gr.4	0	0	
Gr.5	0	0	

^a^Acute xelostomia: Acute toxicity is defined as occurring < 90 days after beginning RT

^b^Toxicity grade was determined according to the Common Terminology Criteria for Adverse Events v3.0 (CTCAE v3.0)

Cox proportional-hazards regression revealed that overall survival was significantly associated with modalities (IGRT versus non-IGRT: HR = 0.46; 95 % CI = 0.22–0.79, *p* = 0.042) and anemia (increasing grade of anemia, HR =2.57; 95 % CI = 1.16–5.67, *p* = 0.020) and that LPFS was significantly associated with modalities (IGRT versus non-IGRT: HR = 0.18; 95 % CI = 0.06–0.56, *p* = 0.003) and anemia (HR = 5.83; 95 % CI = 2.22–15.31, *p* < 0.001) (Table 5).

**Table 5:** Results of Cox proportional hazards analysis after adjusting for modalities, xerostomia, mucositis, dermatitis, body weight loss, dysphagia, fistula formation, anemia, leucopenia and thrombocytopenia

	5-year OS			5-year LPFS		
Adjusted factors	Hazard ratio (HR)	95 % CI	*p* value	Hazard ratio (HR)	95 % CI	*p* value
IGRT vs Non-IGRT	0.46	0.22–0.97	0.042	0.18	0.06–0.56	0.003
^a^Xerostomia (Acute)	0.92	0.45–1.88	0.813	1.32	0.53–3.33	0.554
Mucositis	0.73	0.42–1.27	0.270	0.93	0.41–2.11	0.856
Dermatitis	1.05	0.68–1.63	0.821	0.60	0.32–1.11	0.103
Body weight loss	1.31	0.68–2.54	0.427	0.32	0.09–1.13	0.076
Dysphagia	0.93	0.60–1.45	0.762	0.56	0.27–1.19	0.122
Fistula formation or superficial cases of skin dehiscence	0.00	0.00–0.00	0.978	0.00	0.00–0.00	0.985
Anemia	2.57	1.16–5.67	0.020	5.83	2.22–15.31	0.000
Leucopenia	1.08	0.67–1.73	0.766	0.85	0.47–1.54	0.588
Thrombocytopenia	0.62	0.29–1.33	0.220	0.59	0.23–1.38	0.206

*Abbreviations: CI* confidence Interval, *IGRT* image-guided radiotherapy, *LPFS* local progression-free survival rate, *OS* overall survival rate

^a^Acute xelostomia: Acute toxicity is defined as occurring < 90 days after beginning RT

## Discussion

The long-term overall survival rate among patients with head and neck carcinoma cancer who were treated with standard fractionation combined with concurrent cisplatin was 48 % in the Radiation Therapy Oncology Group (RTOG) 0129 trial [21] and 46 % in the RTOG 9501 trial [16]. Similarly, the 5-year overall survival rate in the non-IGRT group in this study was 47.5 % (Table 2). After adjusting for ECE, PNI, LVSI, two or more positive lymph nodes and T3,4 in the Cox proportional-hazards regression model, we found that patients who underwent IGRT had significantly better OS and LPFS than patients who received IMRT without image guidance (Figs. 2 and 3, Table 3). One possible reason for the striking improvement in overall survival and local disease control outcomes might be attributed to the better target volume dose conformity, homogeneity of daily image-guided HT compared to non-image-guided IMRT [12–14, 22]. More importantly, IGRT allows for daily adjustment in setup error. In fact, this is pointed to by the large number of marginal failures in the non-IGRT group.

Studies have shown that POTT and OTTRT are associated with overall survival and locoregional control. For example, Ang et al. [23] reported that the 5-year overall survival rates and locoregional control rates in patients with advanced head and neck cancer were respectively about 76 % and 38 % lower in patients who completed the package of overall treatment time in > 13 weeks than in those who completed it in < 11 weeks. Similarly, Langendijk et al. [24] reported that high-risk and intermediate-risk OCC patients with a POTT >13 weeks had a worse 3-year locoregional control rate (71 %) than patients with a POTT ≤ 11 weeks (86 %). In addition, Rosenthal et al. [25] found that patients with a POTT <100 days had significantly better overall survival than those who completed the POTT in more than 100 days. Similarly, we also noted that a POTT ≤13 weeks was associated with better 5-year overall survival than a POTT > 13 weeks (Fig. 4a). Additionally, different modalities were found to contribute to the duration of POTT. The number of patients who completed the POTT in < 13 weeks was significantly higher in the IGRT group than in the non-image-guided IMRT group (*p* = 0.001). That finding might explain, at least in part, why the 5-year OS and LPFS rates were higher in the IGRT group than in the non-IGRT group.

In a large review of retrospective data on patients with head and neck cancer who underwent RT, Fowler et al. [26] found that the average rate of loss of locoregional control per week of prolongation of OTTRT was 9 %. Ang and coworkers [23] randomized 151 patients with advanced head and neck cancer to receive either 63 Gy over a 7–week period or 63 Gy over a 5–week period and found that the locoregional control rate was 15 % higher in patients treated for 5 weeks than in those who received therapy for 7 weeks. Langendijk et al. [24] reported that OTTRT >8 weeks resulted in worse 3-year overall survival and locoregional control than OTTRT ≤ 8 weeks in high- and intermediate-risk OCC patients. Moreover, Muriel et al. [27] found that the 5-year locoregional control rate was 75 % in patients with a postoperative irradiation time ≤ 55 days and 68 % in those who received radiation for more than 56 days. In our previous study we showed that around 80 % of patients treated with IGRT could complete the OTTRT within 8 weeks [3]. In this study, we found that patients treated with IGRT were more likely to complete OTTRT within 6.5, 7 or 8 weeks than patients who received non-IGRT. In the current study, patients who completed the OTTRT in ≤ 8 weeks had better 5-year LPFS than those who completed the OTTRT in > 8 weeks (72.3 % *vs.* 40.1 %, *p* = 0.021, Fig. 4b).

Feng and coauthors reported that IMRT results in better swallowing function and a reduced incidence of dysphagia by reducing the dose of RT to pharyngeal constrictors and other swallowing structures [28]. Den et al. [8] also found that IGRT reduces the incidence of dysphagia in patients with head and neck cancer by minimizing the exposure of normal tissues to radiation. Additionally, PTV volumes are important predictors of nutritional compromise and have been used to predict weight loss during radiotherapy in patients with head and neck cancer [29]. Also Capuano et al. [30] found that a reduction in body weight of more than 20 % was significantly correlated with treatment interruption. Furthermore, Arrieta et al. [31] found that the incidence of chemotherapy-induced hematologic toxicity was significantly higher among malnourished patients than among well-nourished patients. With daily MVCT monitoring, the margin of PTV in the IGRT group was smaller than in the non-IGRT group; therefore, the smaller volume in PTV after 3-dimention expansion in the IGRT group could be expected. Markedly more patients in the non-IG-IMRT group than in the IGRT group were unable to complete OTTRT within 7 or 8 weeks. This might explain, at least in part, why a larger PTV margin in the non-image-guided plan influenced the OTTRT. Clinically, there was markedly less grade 2/3 body weight loss in the IGRT group than in the non-IGRT. In addition, the rates of leucopenia and thrombocytopenia were markedly lower in the IGRT group than in the non-IGRT group. After adjusting for adverse factors, we found that IGRT was associated with significantly better OS and LPFS than non-IG-IMRT. (Table 5) Therefore, patients treated with IGRT might have a better chance of completing OTTRT within 8 weeks and hence a better overall outcome.

In our study, the rate of local failure was 24.0 % in the non-IGRT group and 6.8 % in the IGRT group. Among the patients with local failure, the marginal failure rate was 52.6 % in the non-IGRT group and 0 % in the IGRT group. The margin recipe for non-IGRT treatments assumes a statistical 5 % D95 miss in 10 % of the patients [32]. Daily setup variations can range from 3 to 21 %, which will result in unrecognized geographic miss and resultant target underdose [33]. Zeidan et al. [7] found that more than 10 % of patients with head and neck cancer had setup errors of ≥ 5 mm. Moreover, it has been shown that soft tissue shrinkage causes a higher deviation in delivered dose to the PTV and normal tissue outside the PTV [34]. The above-mentioned results suggest that daily image correction minimizes geographic misses and decreases the probability of margin failure. The ranges in variation of registration by daily MVCT for patients treated by IGRT were analyzed retrospectively. The variation in x-axis ranged from 6.4 to−8.7 mm and the variation in the y-axis ranged from 4.7 to −9.7 mm. However, in our institute, the margin for CTV to expand to PTV is 5 mm in non-IGRT system. This may be one of the reasons for the higher marginal failure rate observed in patients treated with non-IGRT. In addition, the rate of weight loss in the non-IGRT group was higher than that in the HT group, which may have led to more deviations in the administered dose distribution with respect to the planned dose, thereby resulting in worse dose coverage and, hence, poorer local control and overall survival. Putting these observations together, it is apparent that a larger margin for CTV to expand to PTV should be considered for non-IGRT.

Studies have shown that patients with head and neck cancer who have inadequate insurance coverage and those with low socioeconomic status have lower rates of survival [35]. In addition, Allareddy et al. [36] found that comorbid conditions and inadequate insurance coverage were predictors of in-hospital mortality. In Taiwan, HT is not covered by the universal National Health Insurance program. Patients who elect to undergo the procedure must either pay out-of-pocket or have a robust private health insurance policy to cover the expenses for the procedure. Therefore, it is assumed that patients who elected to undergo postoperative HT had the economic means to do so.

In vitro studies of conventional photon radiation therapy under normoxic conditions have demonstrated up to three times the efficacy relative to anoxic conditions [37]. Anemia was reported to be an independent prognostic factor in squamous cell carcinoma of the head and neck [38] and a predictive factor for local recurrence in postoperative radiotherapy for head and neck cancer [39]. In this study, we also found that anemia was an independent predictor of poor OS and LPFS (Table 5).

This study has several limitations, most of which are related to its retrospective nature. The patients in our study were treated by a consistent group of radiation, surgical, and medical oncologists; however, these patients were not selected or treated based on a prospective protocol, such as fractionation regimen or simultaneous-integrated or sequential boost techniques, leading to heterogeneity in the management of patients. However, all patients were reviewed by the multidisciplinary tumor board, and hence, all individuals were treated with a consistent treatment philosophy. Second, the choice of treatment modality was left up to the patients. In Taiwan, only IMRT is covered by the National Health Insurance system. Therefore, patients who chose to undergo HT had to pay out-of-pocket for the procedure, which may indicate potential bias. Additionally, the quality of both non-IGRT and IGRT planning improved over time, which is partly evidenced by the elimination of strictures in our later experience. Finally, toxicity data were not prospectively collected but rather abstracted from the medical records. Such a process is limited by the underlying inadequacies of medical documentation when used for research purposes.

## Conclusion

IGRT results in better overall survival and local progression-free survival than IMRT without image guidance in patients with locally advanced OCC. Our results also indicate that patients whose overall duration of IGRT is less than 8 weeks and whose total time from surgery to completion of IGRT is ≤ 13 weeks have better local progression-free survival and overall survival, respectively. Furthermore, there is no significant difference in incidence rates of adverse effects between the two modalities. Finally, our results show that anemia is prognostic of poor outcome in patients with OCC.

## Abbreviations

CCRT: concurrent chemoradiation therapy
CT: computed tomography
CTCAE v3.0: the Common Terminology Criteria for Adverse Events v3.0
CTV: clinical target volume
DFS: disease-free survival
ECE: extracapsular extension
HR: hazard ratio
HT: helical tomotherapy
IGRT: image-guided radiotherapy
IMRT: intensity-modulated radiotherapy
LPFS: local progression-free survival
LRPFS: locoregional progression-free survival
LVSI: lymphovascular space involvement
MFS: metastasis-free survival
MVCT: megavoltage computed tomography
OARs: organs at risk
OCC: oral cavity cancer
OS: overall survival
OTTRT: overall treatment time of radiation therapy
PNI: perineural involvement
POTT: package of overall treatment time
PTV: planning target volumes
RPFS: regional progression-free survival
RTOG: Radiation Therapy Oncology Group
TNM: tumor-node-metastasis
